# Comparative genomics reveals cellobiose hydrolysis mechanism of *Ruminiclostridium thermocellum* M3, a cellulosic saccharification bacterium

**DOI:** 10.3389/fmicb.2022.1079279

**Published:** 2023-01-06

**Authors:** Sheng Tao, Meng Qingbin, Li Zhiling, Sun Caiyu, Li Lixin, Liu Lilai

**Affiliations:** ^1^College of Environmental and Chemical Engineering, Heilongjiang University of Science and Technology, Harbin, China; ^2^State Key Lab of Urban Water Resource and Environment, Harbin Institute of Technology, Harbin, China

**Keywords:** thermocellum, genome, cellobiose, **β**-glucosidase, CAZyme

## Abstract

The cellulosome of *Ruminiclostridium thermocellum* was one of the most efficient cellulase systems in nature. However, the product of cellulose degradation by *R. thermocellum* is cellobiose, which leads to the feedback inhibition of cellulosome, and it limits the *R. thermocellum* application in the field of cellulosic biomass consolidated bioprocessing (CBP) industry. In a previous study, *R. thermocellum* M3, which can hydrolyze cellulosic feedstocks into monosaccharides, was isolated from horse manure. In this study, the complete genome of *R. thermocellum* M3 was sequenced and assembled. The genome of *R. thermocellum* M3 was compared with the other *R. thermocellum* to reveal the mechanism of cellulosic saccharification by *R. thermocellum* M3. In addition, we predicted the key genes for the elimination of feedback inhibition of cellobiose in *R. thermocellum*. The results indicated that the whole genome sequence of *R. thermocellum* M3 consisted of 3.6 Mb of chromosomes with a 38.9% of GC%. To be specific, eight gene islands and 271 carbohydrate-active enzyme-encoded proteins were detected. Moreover, the results of gene function annotation showed that 2,071, 2,120, and 1,246 genes were annotated into the Clusters of Orthologous Groups (COG), Gene Ontology (GO), and Kyoto Encyclopedia of Genes and Genomes (KEGG) databases, respectively, and most of the genes were involved in carbohydrate metabolism and enzymatic catalysis. Different from other *R. thermocellum*, strain M3 has three proteins related to β-glucosidase, and the cellobiose hydrolysis was enhanced by the synergy of gene *BglA* and *BglX*. Meanwhile, the GH42 family, CBM36 family, and AA8 family might participate in cellobiose degradation.

## 1. Introduction

Lignocellulosic biomass was considered an ideal sustainable resource for potential feedstock for various value-added chemicals and biofuels (Khare et al., 2015; Usmani et al., 2021). As a result, the rational use of lignocellulosic biomass not only relieves the pressure of fossil energy shortage but also mitigates natural environment damage caused by improper treatment (Staples et al., 2017). Converting lignocellulosic feedstocks into high-value chemical products includes three steps: pretreatment, saccharification, and fermentation (Yadav et al., 2020). Saccharification refers to the hydrolysis of holocellulose into monosaccharides/oligosaccharides, which facilitates the downstream process. The saccharification of lignocellulose feedstocks was one of the bottlenecks of lignocellulosic biomass utilization (Guo et al., 2018; Usmani et al., 2020). Cellulosic biomass can be saccharified by acids or cellulases. Cellulose hydrolysis by cellulase is considered more environmentally friendly than cellulose hydrolysis by acid when conducted under mild conditions. The degradation of cellulose is achieved by the synergistic action of endoglucosidase, extranosidase, and β-glucosidase (Sheng et al., 2016). Both bacteria and fungi can synthesize cellulase in nature; *Trichoderma* and *Aspergillus* sp. are known for their potential to produce cellulases, while these fungi lack a complete cellulase system, which leads to a decrease in the catalytic efficiency of cellulase (Srivastava et al., 2018). Anaerobic bacteria degrade cellulose by synthesizing cellulosomes, which gather different cellulases in a narrow space and anchor them on the cell surface, the cellulosome of *R. thermocellum* is the most efficient cellulase system found at present (Mazzoli and Olson, 2020).

However, the hydrolysis of lignocellulosic biomass by *R. thermocellum* has not been industrialized for the low activity of β-glucosidase, which is insufficient for the lignocellulosic feedstocks saccharification (Sheng et al., 2016). Moreover, the feedback inhibition of exoglucanosidases induced by the accumulation of cellobiose severely reduced the catalytic efficiency of the cellulosome (Lamed et al., 1985; Tian et al., 2016; Haldar and Purkait, 2020). The addition of exogenous β-glucosidase is a way to improve the hydrolysis efficiency of cellulosome, but additional β-glucosidase directly increases the cost and complexity of the saccharification process. Accordingly, enhancing the activity of β-glucosidase activity of wild *R. thermocellum* by building recombinant strains that secrete large amounts of β-glucosidase was a promising solution. Meki et al. fused the *E. coli* plasmid containing cloned bglA into wild *R. thermocellum* ATCC 27405 to construct a recombinant strain *R. thermocellum* ATCC 27405 (+*McbglA*), the result indicated that the β-glucosidase activity expressed by recombinant strain was 2.3 times higher than that of the wild strain at the late logarithmic growth stage (Maki et al., 2013). Waeonukul et al. found that the addition of *BglB* from *R. thermocellum* S14 to cellulosome was observed to increase the saccharification rate of cellulose compared to that of Novazyme-188 and cellulosome alone (Waeonukul et al., 2012). Nevertheless, the activity and stability of β-glucosidase secreted by recombinant strain decreased during the hydrolysis process (Zhang et al., 2017). Isiam et al. found that when cellobiose was used as a carbon source, seven cellulosome structural proteins, 31 cellulosome-related glycosidases, and 19 non-cellulosome glycoside hydrolases were expressed in *R. thermocellum*, which suggests that the degradation of cellobiose by *R. thermocellum* was not only related to the β-glucosidase gene but also associated with genes other than β-glucosidase (Islam et al., 2006). Therefore, it is particularly important to find strains with a stable ability to degrade cellobiose and reveal the genes involved in stable cellobiose degradation by *R. thermocellum*.

In previous studies, we isolated an *R. thermocellum* M3 that can efficiently degrade lignocellulosic biomass from horse manure. Different from other *R. thermocellum*, 97% of the cellulosic saccharification products of *R. thermocellum* M3 were monosaccharides. More importantly, *R. thermocellum* M3 inherited the ability of cellobiose degradation stably that conducts *R. thermocellum* M3 being an excellent sample for stable expression of exogenous β-glucosidase in the genus of *R. thermocellum*. In this study, we report the whole genome sequence of *R. thermocellum* M3 and compare the high-quality complete genome sequence of *R. thermocellum* M3 with both intra- and inter-generically to those of its close or distant phylogenetic relatives. Moreover, genes related to cellobiose degradation were comprehensively analyzed.

## 2. Materials and methods

### 2.1. Bacterial strain and cultivation

*Ruminiclostridium thermocellum* M3 strain was isolated and enriched from horse manure by Sheng et al. (2016) and deposited in the Microbiology Laboratory of the School of Environment and Chemical Engineering, Heilongjiang University of Science and Technology. The seed was stored in a constant temperature incubator at −20°C and cultivated in an anaerobic bottle (filled with nitrogen) containing modified ATCC 1191 (MA) medium. The main components of culture medium were K_2_HPO_4,_ 1.5 g/L; MgSO_4_·7H_2_O, 0.2 g/L; (NH_4_)_2_SO_4,_ 1.0 g KCl, 0.2 g/L; L-cysteine, 0.5 g/L; KH_2_PO_4,_ 3.0 g/L; CaCl_2_•2H_2_O, 0.025 g/L; NaCl, 1.0 g/L; Yeast, 1.5 g/L; and Avicel, 5.0 g/L. The temperature of the culture was maintained at 60°C at 120 rpm.

### 2.2. DNA extraction and whole genome sequencing

The *R. thermocellum* samples for whole genome sequencing analysis were cultured in MA medium for 24 h at 60°C, centrifuged for 5 min at 4°C, 12,000 × *g*, then the cell pellet was washed twice with normal saline (NS), and crushed with the FastPrep-24 instrument in lysing matrix B tubes (MP Biomedical) for 40 s to release the genomic DNA from cells. The extraction of genomic DNA was performed with the Tiangen bacterial DNA mini kit (Tiangen Biotech Co. Ltd., Beijing, China) according to the manufacturer’s protocol. The harvested DNA was detected using agarose gel electrophoresis and then quantified by Qubit 4.0 (ThermoFisher, Q33226). The genomic sequencing was conducted by the Pacbio sequencing platform with *de novo* assembly (SMRT portal; Berlin et al., 2015).

### 2.3. Genome annotation and component prediction

The genome annotation and gene function were predicted in the Gene Ontology (GO) database, the Kyoto Encyclopedia of Genes and Genomes (KEGG) database, the Clusters of Orthologous Groups (COG) database, and the Non-Redundant Protein databases (NR). The whole genome BLAST search (E-value below 1^e−5^, minimal alignment length percentage above 40%) was performed with the above four databases. The prediction of carbohydrate-active enzymes was conducted with the Carbohydrate-Active enZYmes Database (Lombard et al., 2013).

Genome component prediction of *R. thermocellum* M3 strains included the coding gene, repetitive sequences, signal peptide, genomic islands, prophage, lipoprotein, prophage, and clustered regularly interspaced short palindromic repeat sequences (CRISPR). The GeneMarkS program was conducted to retrieve the coding genes (http://topaz.gatech.edu/; Yang et al., 2020). The tRNA was predicted by the Aragorn program (Laslett and Canback, 2004), the rRNA was predicted by the RNAmmer program (Lagesen et al., 2007), and the miscRNA was predicted by Infernal (v1.1.2; Mostajo Berrospi et al., 2019). Repeat Modeler was used to predict the repeat sequence *de novo* of the assembly results, and the RepeatMasker program was used for identifying repetitive elements in nucleotide sequences.

Genomic islands were predicted by the Island Path-DIOMB program (Hsiao et al., 2003). The CRISPR was predicted by the CRISPR recognition tool (CRT; Grissa et al., 2007). Prophages were predicted using PhiSpy (Hähnke et al., 2009). RepeatMasker was used to identify the location and frequency of repeats on the genome (Saha et al., 2008). NCBI Blast+ was used to compare the protein sequences with CDD, KOG, COG, NR, NT, PFAM, Swissprot, TrEMBL, and other databases to obtain the functional annotation information.

### 2.4. Whole genome-based comparative genomic analysis

The core genes, specific genes, the gene family phylogenetic tree, single nucleotide polymorphism (SNP), and genome visualization were analyzed to reveal the result of comparative genomics (Zhong et al., 2018). The genome sequences of *R. thermocellum* DSM 2360, *R. thermocellum* ATCC 27405, *R. thermocellum* DSM 1313, and *R. thermocellum* AD2 were obtained from the NCBI database. Genomic alignments among *R. thermocellum* M3 and other genomes of *R. thermocellum* were performed using the MUMmer and LASTZ tools (Kurtz et al., 2004). Core genes and specific genes were analyzed using the CDHIT rapid clustering of similar proteins software with a threshold of 50% pairwise identity and a 0.7 length difference cutoff for amino acids (Li et al., 2001, 2002; Li and Godzik, 2006). The relationships between five *R. thermocellum* strains were analyzed, and the results were represented in a Venn diagram. NCBI Blast+ was used to compare the predicted 16S rRNA sequence with the NCBI 16S database to obtain its homologous strain information and a phylogenetic tree was constructed using mega software (Hall, 2013).

## 3. Results

### 3.1. Feature of the whole genome of *Ruminiclostridium thermocellum* M3 genome

The whole genome of *R. thermocellum* M3 was sequenced and analyzed with regard to the predictions of coding genes. The identified total size of the genome *R. thermocellum* M3 was 3,602,270 bp with 39% GC content using SPAdes, and the number of coding genes was 3,195 with an average gene length of 973.56 bp (Supplementary Figure S1). The major characteristics of the genome of the *R. thermocellum* M3 and four *R. thermocellum* are summarized to acquire more perceptions concerning the genetic information (Table 1). The final assemblies indicated that the genomes of five *R. thermocellum* were similar in size and G + C contents, genome annotation yielded 3,062, 3,196, 2,949, 2,959, and 3,077 genes for strains DSM 2360, ATCC 27405, DSM 1313, AD2, and M3, respectively.

**Table 1:** General features of *Ruminiclostridium thermocellum* M3 genome and comparison with other closely related species.

Features	DSM 2360	ATCC 27405	DSM 1313	AD2	M3
Genome Size (Mb)	3.57758	3.8433	3.56162	3.55485	3.60227
G + C content (%)	39.2	39	39.1	39.2	38.9
No. of Scaffolds	1	1	1	1	1
No. of CDS	3,026	3,196	2,949	2,959	3,077
No. of rRNA operons	12	12	12	12	12
No. of tRNA operons	56	56	56	56	56
ANI (%)	99.65	99.65	99.58	99.65	100

**Figure 1:**
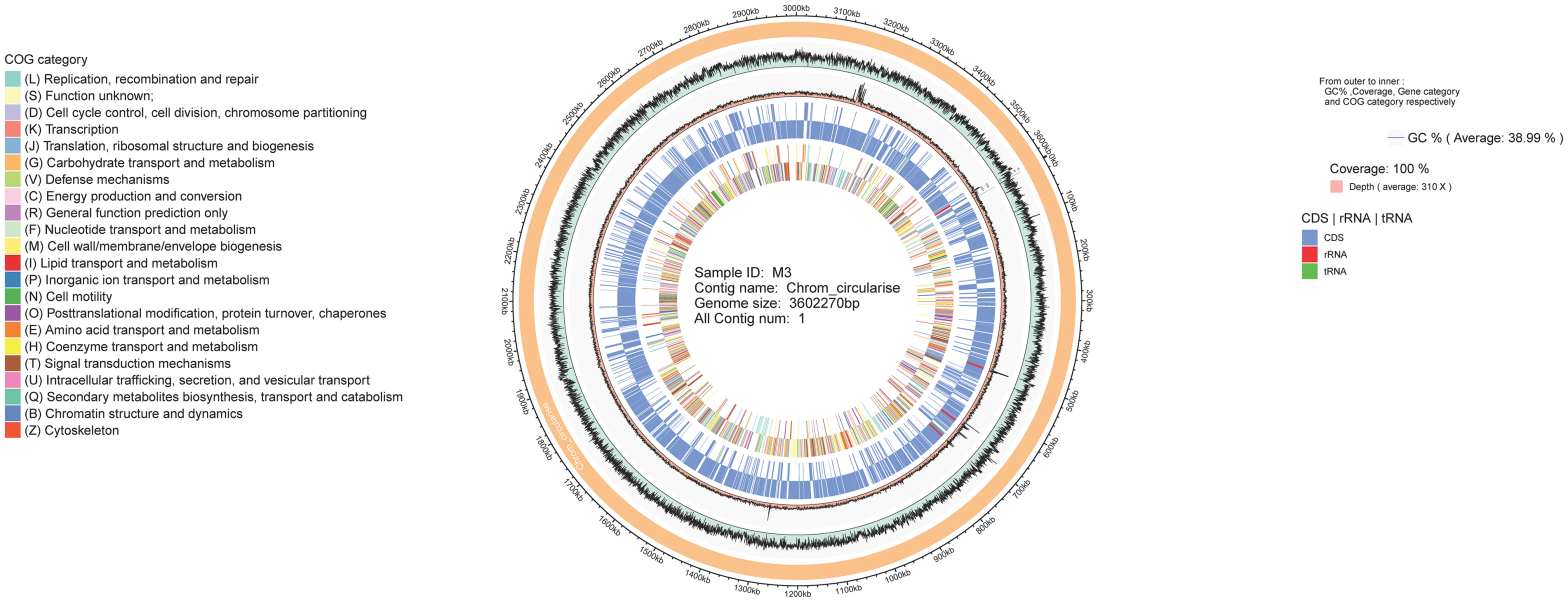
Whole-genome visualization map of *Ruminiclostridium thermocellum* M3.

The result of genome annotation indicated that a series of genes encoding virulence factors (VFDB), antibiotic resistance (CARD), and pathogen-host interactions (PHI-base) were present in *R. thermocellum* (Supplementary Table S1). Meanwhile, *R. thermocellum* strains had several genes related to carbohydrate degradation, biosynthesis, and modification enzymes. The whole-genome visualization map clearly identified the composition, location, and function of the *R. thermocellum* M3 genome, which is demonstrated in Figure 1, with 100% coverage of sequencing. Detailed information includes analysis of CDS, non-coding RNA, COG, functional classification of genes, and the size of genome and GC%. Additionally, among the 3,077 predicted genes of *R. thermocellum* M3, only 2,071 CDSs were assigned any COG, which accounted for 67.3% of the predicted genes, and an additional 171 CDSs were assigned to group S with an unknown function. There are 137 genes involved in amino acid production and transformation (group E) in the aligned COG database genes, accounting for 6.62% of the COG annotations; the number of carbohydrate transport and metabolism-related genes (group G) was 130, accounting for 6.28% of the COG-annotated genes; the quantity of energy production and conversion-related genes (group C) was 107, taking up 5.17% of the COG-annotated genes; and only 16 genes fall into group Q (secondary metabolites biosynthesis, transport, and catabolism; see Supplementary Figure S2).

### 3.2. Genome functional studies of *Ruminiclostridium thermocellum* M3

Gene Ontology and KEGG databases were used to obtain the elucidation of the “character” of the coding gene in the bacteria from a macroscopic perspective. In the genome of *R. thermocellum* M3, 2021 genes were annotated in the GO database (Table 2). In the biological process, the metabolic and cellular processes account for the highest proportion; in molecular function, it is mainly related to catalytic activity and binding; in the cellular component, it is closely compared to cells and cell parts, which indicated that strain M3 has more proteins involved in metabolism, cell composition, and enzymatic catalysis. These results are in accordance with the biochemical characteristics of M3, which possesses an outstanding catalytic capacity for cellulose substrate. In the KEGG database, 1,246 genes were annotated, which can be divided into five branches according to the metabolic pathways involved in the genes: cellular processes, environmental information processing, genetic information processing, metabolism, and organismal systems. As shown in Supplementary Figure S3, aligning these annotated genes into metabolic pathways yielded a total of 147 metabolic pathways. It can be clearly seen that the predominant pathway was carbohydrate metabolism and overview with 329 and 229 unigenes, followed by amino acid metabolism and energy metabolism with 197 and 153 unigenes.

**Table 2:** Gene function analysis of *Ruminiclostridium thermocellum* M3 based on Gene Ontology (GO) annotation.

Ontology	GO-ID	Term	Gene-Num	Ratio
Molecular function	GO:0003824	Catalytic activity	1,432	46.54
	GO:0005488	Binding	1,293	42.02
	GO:0005215	Transporter activity	147	4.78
	GO:0060089	Molecular transducer activity	106	3.44
	GO:0001071	Nucleic acid binding transcription factor activity	73	2.37
Cellular component	GO:0005623	Cell	1,035	33.64
	GO:0044464	Cell part	1,035	33.64
	GO:0016020	Membrane	482	15.66
	GO:0044425	Membrane part	363	11.8
	GO:0043226	Organelle	141	4.58
	GO:0032991	Macromolecular complex	118	3.83
Biological process	GO:0008152	Metabolic process	1,468	47.71
	GO:0009987	Cellular process	1,419	46.12
	GO:0044699	Single-organism process	597	19.4
	GO:0050896	Response to stimulus	313	10.17
	GO:0065007	Biological regulation	245	7.96
	GO:0050789	Regulation of biological process	235	7.64
	GO:0051179	Localization	200	6.5
	GO:0051234	Establishment of localization	176	5.72

Meanwhile, the predicted numbers of SignaIP-TM and SignaIP-noTM were 24 and 106, respectively, among the total Signa proteins. A total of four CRISPR arrays were found by CRT (Supplementary Table S2). The number of repeat counts in four arrays was 51, 90, 134, and 144. Moreover, several cas or cas-like genes were found in their neighborhoods. This suggests that R*. thermocellum* M3 has a defense against phage contamination, as CRISPR is very important in prokaryotes and is involved in resisting foreign phages and plasmids and recognizing and silencing invading functional elements. The results of gene-island (GI) prediction obtained eight GI with an average G + C content of about 36.4%, which is slightly lower than the G + C content of the M3 genome. It is worth noting that the G + C content of GI5 (33.7%) is significantly different than that of other GI, which indicates GI5 may be an exogenous sequence by horizontal transfer. The main components of the exogenous sequence are the transposase protein and the hypothetical protein (Supplementary Table S3).

### 3.3. Comparative genomics of *Ruminiclostridium thermocellum*

**Figure 2:**
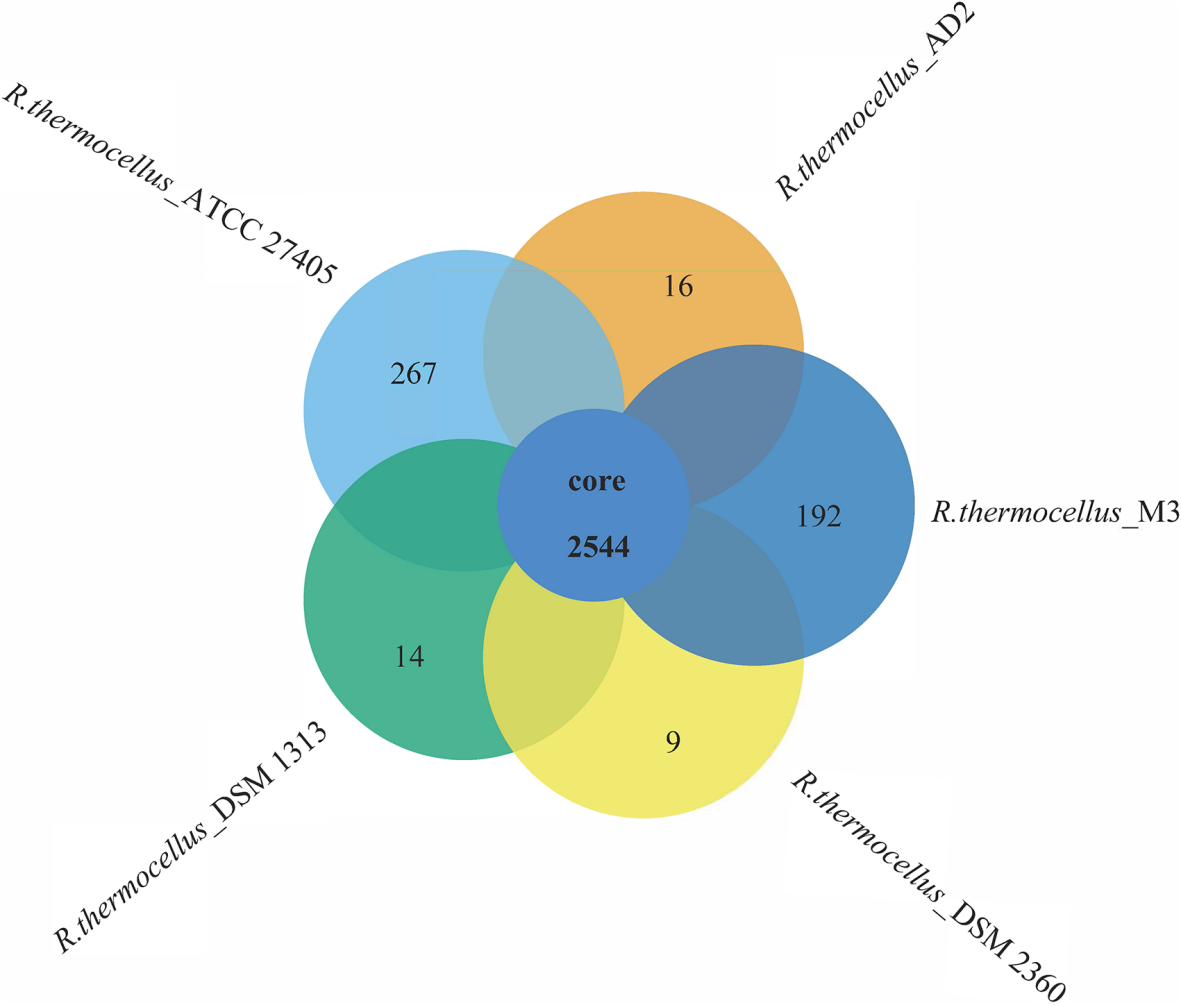
Venn diagram of core and specific genes among five *R. thermocellum* strains. Each circle represents an *Ruminiclostridium thermocellum* strain. The number of orthologous coding sequences (core genome) shared by all strains is shown in the center circle, and the number of specific genes is shown in non-overlapping portions of each oval.

To reveal the genetic and evolutionary relationships between *R. thermocellum* M3 and other typical *R. thermocellum*, the analysis in view of the core-pan gene of the whole genome sequence was conducted. The Gene family boxplot (Supplementary Figure S4) revealed that the pan-genome trend of *R. thermocellum* is open with the increasing number of *R. thermocellum* strains sequenced. The open pan-genome indicated that there is a significant capacity for discovering novel genes with the evolution and development of strains. Among the five *R. thermocellum* strains, the number of genes in the pan-genome is 3,042 and includes a core gene set (2,544 genes; Figure 2). Dispensable genes and unique genes existed across all genomes of five *R. thermocellum* strains. Among them, strain ATCC 27405 had the largest number of specific genes (267 genes), followed by strain M3 (192 genes). The high resemblance of five *R. thermocellum* strains was reflected by the large proportion of core genes.

**Figure 3:**
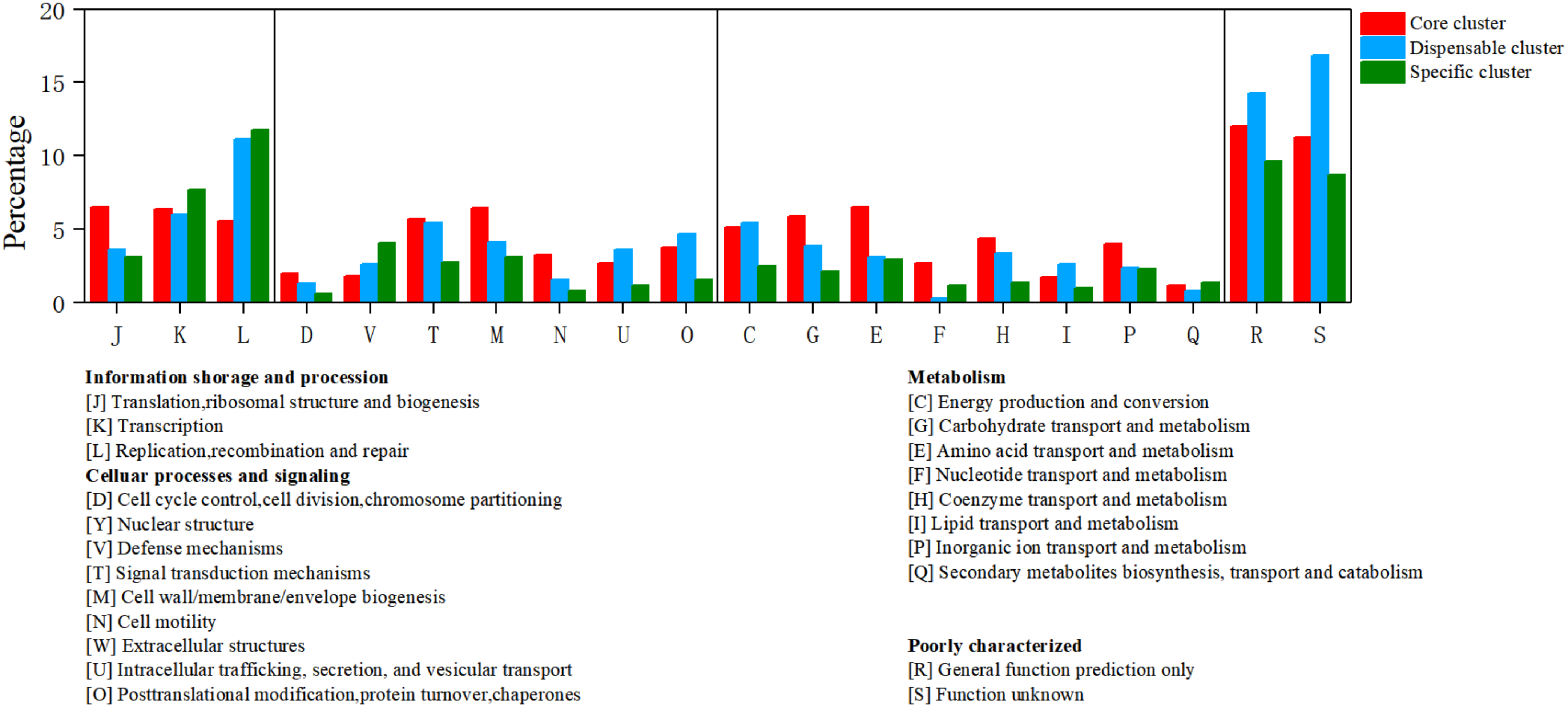
Distribution of core, dispensable, and specific genes on the Clusters of Orthologous Groups (COG) category of *Ruminiclostridium thermocellum* M3.

**Figure 4:**
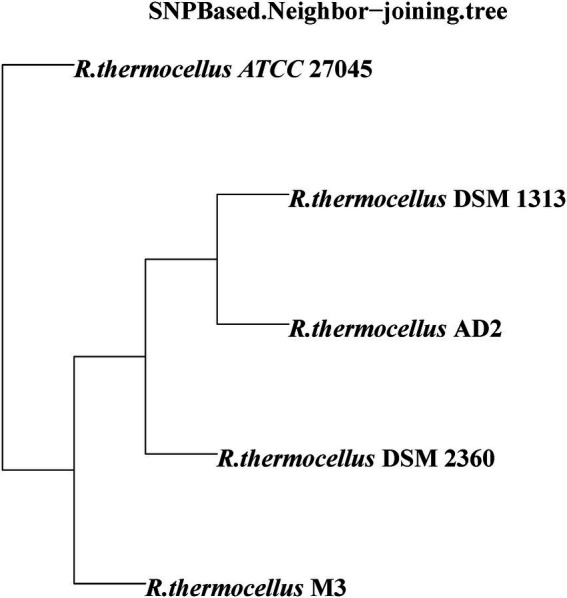
Phylogenetic relationship of *Ruminiclostridium thermocellum* M3 and other four *R. thermocellum* strains. Numbers along branches indicate bootstrap values with 1,000 times.

**Figure 5:**
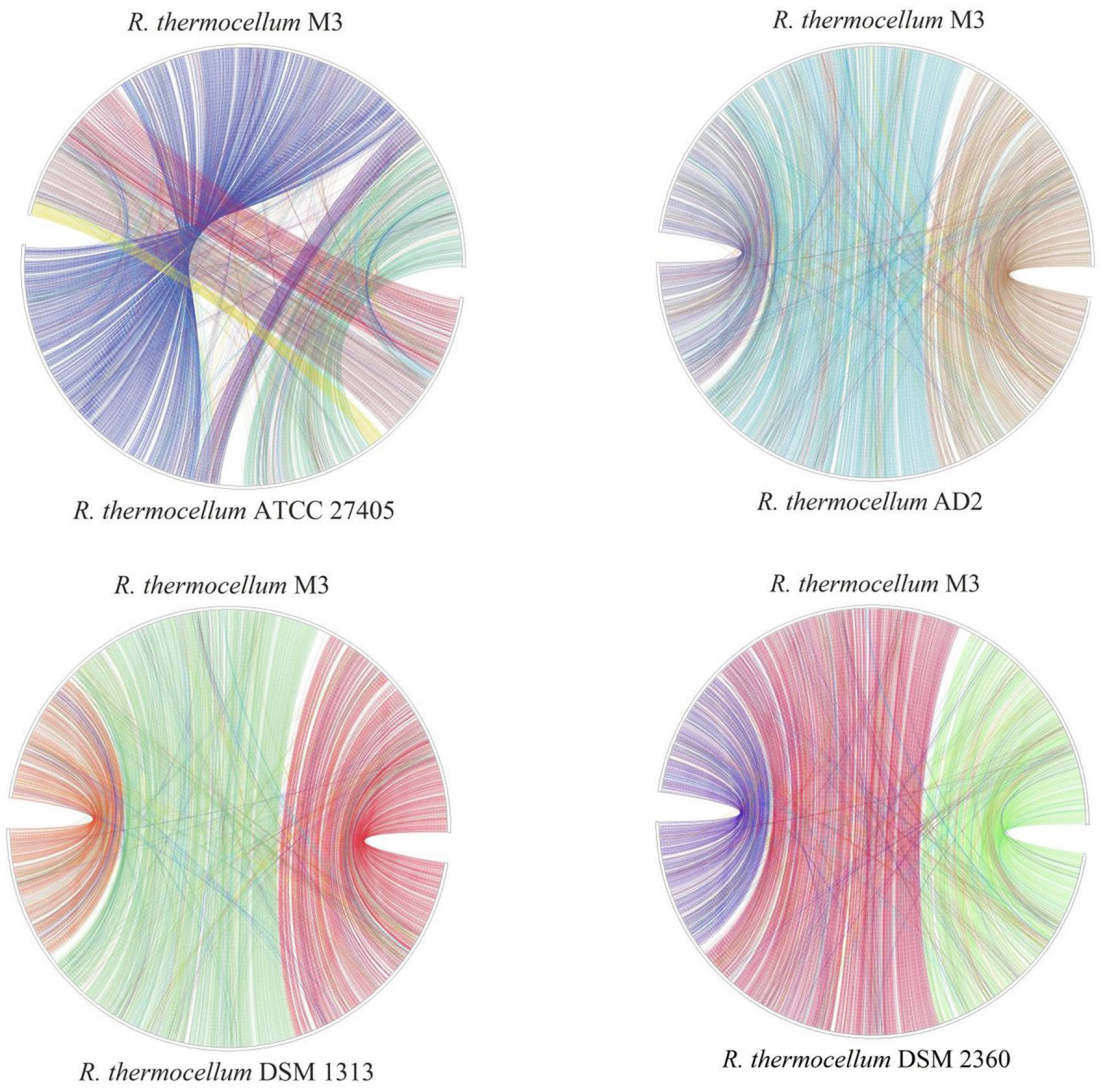
Plot of protein linear analysis between *Ruminiclostridium thermocellum* M3 and other four *R. thermocellum.*

To identify the functional classes of the *R. thermocellum* pan-genome, the COG database was used to classify the functional genes. In the pan-genome of *R. thermocellum*, the most core, dispensable, and specific gene clusters fell in the metabolism category (Figure 3). Meanwhile, the results indicated that both gene clusters for general function prediction and unknown functions were also more abundant. Compared with the dispensable and specific gene clusters, the majority of genes in the core gene clusters were involved in translation, ribosomal structure, biogenesis (J), cell wall/membrane/envelope biogenesis (M), carbohydrate transport and metabolism (G), amino acid transport, and metabolism (E). By comparison, the majority of genes in the no-core gene clusters was concerned with housekeeping functions, for example, replication, recombination and repair (L), and defense mechanisms (V). Single nucleotide polymorphism (SNP) represents the variation situation of bacteria in the evolutionary process. We constructed the phylogenetic tree based on SNP, which revealed the similarity in bacterial strain variation in adaptation to the natural environment. Compared with *R. thermocellum* ATCC 27405, M3 showed stronger evolutionary relationships with the other three *R. thermocellum* strains (Figure 4). Orthologous protein linear analysis based on MCScanX software was performed to further understand the differences in protein homology and amino acid arrangement between M3 and the other four *R. thermocellum* strains (Figure 5). Between M3 and other three *R. thermocellum* strains (DSM 1313, DSM 2360 and AD2), it was found that the proteins are not only homologous but also have a good linear relationship in sequence, while *R. thermocellum* ATCC 27405 was related to M3 but showed a large number of inversions. At the same time, we performed a genome-linear analysis of M3 and *R. thermocellum* ATCC 27405 (Supplementary Figure S5). The results were similar to the results of the orthologous protein analysis, and it was more clearly evident that nearly half of the genes were inversions.

### 3.4. Protein-encoding genes related to the CAZyme system of *Ruminiclostridium thermocellum M3*

The results of the CAZyme system analysis indicated that the multi-modular enzyme system of *R. thermocellum* M3 consisted of 75 dockerin and eight cohesin, which construct scaffolding structural proteins offering a large number of binding sites for cellulase. The quantity of enzyme protein and composition across dissimilar CAZy families in *R. thermocellum* M3 were analyzed and compared to those in the other four *R. thermocellum* to evaluate the inclination for lignocellulose saccharification. In the genome of *R. thermocellum*, most genes fell into glycoside hydrolases (GHs), carbohydrate binding molecules (CBMs), and glycosyltransferases (GTs), whereas a few genes were annotated in carbohydrate esterases (CEs), polysaccharide lyases (PLs), and auxiliary activities (AAs). To be specific, 271 cellulase proteins were detected in *R. thermocellum* M3 using dbCAN2 (DIAMOND algorithm; Table 3). Most proteins were detected to be GHs (108 candidates), with GH124 (*n* = 71), GH9 (*n* = 17), and GH5 (*n* = 13) being the most abundant families. In addition, there were 75 carbohydrate-binding module (CBM) proteins, 55 glycosyl transferases (GTs) proteins, 22 carbohydrate esterases (CEs) proteins, seven polysaccharide lyases (PLs) proteins, and four auxiliary activities (AAs) proteins. It is worth noting that there were nine CBM genes and 24 GH genes that had not been identified in *R. thermocellum* before (Supplementary Table S4).

**Table 3:** Comparison of the number of enzyme protein in *Ruminiclostridium thermocellum* M3 and other *R. thermocellum*.

	GH	CBM	GT	CE	PL	AA	Total
*R. thermocellum* DSM 2360	108	72	55	22	8	4	269
*R. thermocellum* ATCC 27405	102	70	56	22	7	3	260
*R. thermocellum* DSM 1313	110	75	54	22	8	4	273
*R. thermocellum* AD2	108	72	55	22	8	4	269
*R. thermocellum* M3	108	75	55	22	7	4	271

GH, glycoside hydrolase; GT, glycoside transferase; AA, auxiliary activity; CE, carbohydrate esterase; PL, polysaccharide lyase; and CBM, carbohydrate-binding module. All CAZy domains were identified using dbCAN2 (DIAMOND algorithm).

**Figure 6:**
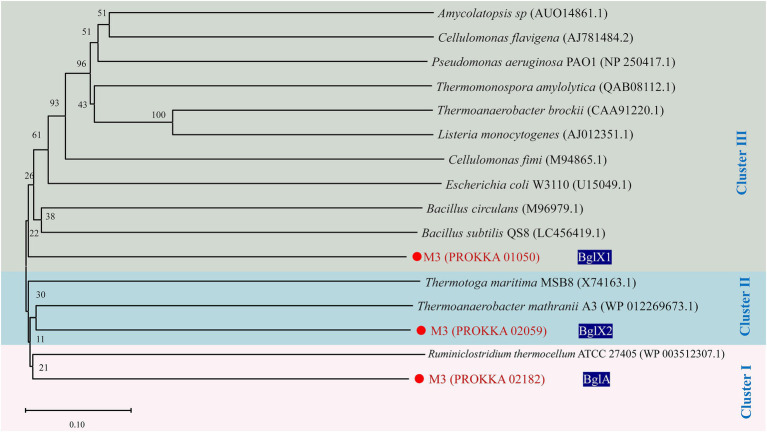
Phylogenetic tree of β-glucosidase from *Ruminiclostridium thermocellum* M3. Numbers along branches indicate bootstrap values with 1,000 times.

Four encoded proteins associated with cellobiose degradation were found among the cellulase-encoded proteins of strain M3 by Conserved Domain Database (CDD) analysis (Supplementary Figure S6), including *BglA* (Protein: PROKKA_02182), *BglB* (Protein: PROKKA_00862), and *BglX* (Protein: PROKKA_01050 and PROKKA_02059). *BglA* encoded glycoside hydrolase family 1 (GH1) protein (β-glucosidase). *BglB* encoded glycoside hydrolase family 1 (GH1) protein (6-phospho-β-glucosidase). *BglX* (PROKKA_01050) encoded glycoside hydrolase family 3 (GH3) protein (β-glucosidase). *BglX* (PROKKA_02059) encoded glycoside hydrolase family 3 (GH3) protein (β-glucosidase). The result of protein sequence alignment indicated that the ratio of similarity of pairwise comparison of proteins encoded by three genes (PROKKA_02182, PROKKA_01050, and PROKKA_02059), which correlated with β-glucosidase, were lower than 28.39%. To further demonstrate the diversity among the three *Bgls*, we constructed phylogenetic trees of *Bgls* from different strains by MEGA software. Not surprisingly, the phylogenetic tree based on β-glucosidase showed that each of the three *Bgls* was located in one of three different clusters (Figure 6). Furthermore, a gene encoding β-galactosidase (PROKKA_03012) was present in the M3 genome, which was not found in the other wild *R. thermocellum* before.

In addition, 11 coding proteins about ABC sugar transport protein and two coding proteins (PROKKA_01086 and PROKKA_02112) about cellobiose phosphorylase coming from glycoside hydrolase family 94 (GH94) were detected in the genome of *R. thermocellum* M3 (Supplementary Table S5). Two group coding proteins (MglA and UpgA) relating polysaccharide transportation (cellobiose, fructose, and arabinose) ware found in 11 ABC sugar transport proteins by CDD. MglA (PROKKA_00080 and PROKKA_01980) is the ATPase component of ABC sugar transporter, which mainly promotes sugar transport; the primary function of UpgA (PROKKA_01330 and PROKKA_02933) is the transmembrane of sugar substrates, which is a permease component of the ABC transporter. Meanwhile, two unique ABC sugar transport proteins (PROKKA_02931 and PROKKA_02932), which had not been reported in other *R. thermocellum*, were found in strain M3.

## 4. Discussion

### 4.1. Genome specificity of cellobiose hydrolysis by *Ruminiclostridium thermocellum* M3

*Ruminiclostridium thermocellum*, as a typical genus of thermophagic cellulosic-degrading bacteria, was a high-utility candidate in lignocellulosic biomass refinement by means of a powerful cellulosome system (Akinosho et al., 2014; Sheng et al., 2016). However, the activity of β-glucosidase (*BglA*) was low in wild-type *R. thermocellum* strains (Shinoda et al., 2019), and due to the adsorption of cellulosome to cellulose, only a small fraction of *BglA* was available on cellulosome. In nature, the efficient decomposition of cellulosic biomass strongly depends on the fast adaptation of *R. thermocellum* to enable the regulation of its cellulosomal enzymes for a specific substrate composition. Compared to other *R. thermocellum*, such as genus *R. thermocellum* ATCC 27045 and AD 2, genus *R. thermocellum* M3 not only has high cellulosic saccharification ability but also possesses the properties of cellubiose-resistance, which contributes to M3 being a potential industrial option for cellulosic biofuels refine.

In general, the wild type *R. thermocellum* containing β-glucosidase A (*BglA*) and β-glucosidase B (*BglB*), gene *BglX* (β-glucosidase) was first found in *R. thermocellum* in this study. In general, β-glucosidase A was sensitive to temperature and thus inactivated under the optimum temperature of *R. thermocellum* (Yoav et al., 2019). Different from β-glucosidase A, *BglB* encodes a novel thermostable β-glucosidase B, which is more thermally stable than β-glucosidase A, whereas the biosynthesis of β-glucosidase B is repressed by cellobiose. Moreover, the β-glucosidase B of *R. thermocellum* was inhibited by low glucose concentration (Waeonukul et al., 2012). Different from *BglA* and *BglB*, the gene *BglX* (Protein:PROKKA_01050) may encode β-xylosidase or β-glucosidase, since *R. thermocellum* cannot utilize xylose as a carbon source as the previous report (Sheng et al., 2016) suggests that the *BglX* (Protein: PROKKA_01050) gene mainly plays a role in the hydrolysis of cellobiose in strain M3 and that the *BglX* (PROKKA_01050) gene encodes an enzyme with β-glucosidase activity predominantly. It is reported that the β-glucosidase encoded by *BglX* was a periplasmic cellulase that hydrolysis cellobiose into glucose (Souto et al., 2021). In addition, a signal peptide that anchors β-glucosidase *BglX* to the periplasmatic space was found in the conserved domain of *BglX*, which might help to reduce the cellobiose concentration in a restricted area of the cell surface (Souto et al., 2021). Kim also found that the β-glucosidase of *Aspergillus aculeatus* can bind to the yeast surface without any modification (Kim et al., 2013), which suggests that the β-glucosidase of strain M3 could be directly connected to the cell membrane to hydrolyze cellobiose without the involvement of the CBM module.

It is believed that the genome of *R. thermocellum* does not encode any β-galactosidase (GH42; Ravachol et al., 2016). Surprisingly, GH42 β-galactosidase (PROKKA_03012) was found in the genome of *R. thermocellum* M3. Some studies found that in addition to β-glucosidase, β-1,4 glucosidic can also be cleaved by β-galactosidase (Morita et al., 2008; Yang et al., 2018). Therefore, GH42 might be another key factor in cellobiose hydrolysis by *R. thermocellum* M3. Similar to GH42, GH116 was not found in *R. thermocellum* in the previous study; the GH116 family was reported as a thermophilic β-d-glucosidase, which was found in animals, plants, archaea, and bacteria (Sansenya et al., 2015; Rohman et al., 2019). The GH116 protein from thermophilic bacterial was reported for high hydrolytic activity toward β-1,3- and β-1,4-linked gluco-oligosaccharides and 4-nitrophenyl β-D-glucopyranoside (4NPGlc) artificial substrate (Sansenya et al., 2015); therefore, GH116 protein might be another key factor involved in the hydrolysis of cellobiose by *R. thermocellum* M3.

The combination of ABC transport protein and cellobiose phosphorylase is a common strategy concerning cellobiose transportation in *R. thermocellum* (Parisutham et al., 2017). Five putative cellodextrin-specific ABC transporters, labeled as CbpA-D and Lbp, had been identified in *R. thermocellum* DSM 1313 (Nataf et al., 2009); Yan et al. found that only CbpB plays a key role in cellobiose transport using the functional verification of CbpA-D by genetic inactivation (Yan et al., 2022). We identified four ABC sugar transporters that specifically transport polysaccharides by gene annotation and conserved domain database analysis, but the high affinity for binding to cellobiose needs further proof in future works.

### 4.2. Genome specificity of carbohydrate binding module of *Ruminiclostridium thermocellum*

It is believed that the cellulase system of *R. thermocellum*-cellulosomes contains cellulose-binding modules (CBMs), which leads to catalytic activity varying greatly in different regions. As a result, there are higher local cellobiose concentrations at particular sites. However, the β-glucosidase of wild type *R. thermocellum* was not bound to the CBM which led to the ineffective hydrolysis of local cellobiose (Yoav et al., 2019). Therefore, the construction of an artificial chimeric cohesion-containing scaffold in which binding the β-glucosidase to the cellulosome and mimicking the enzymatic synergism of native cellulosome systems was feasible to enhance the hydrolysis efficiency of cellobiose (Fierobe et al., 2002, 2005; Morais et al., 2010). Different from other *R. thermocellum* strains, nine CBM family genes were unique to strain M3, including CBM23, CBM36, CBM37, CBM40, CBM47, CBM53, CBM61, CBM70, and CBM75. It is worth noting that the CBM37s were initially discovered in *R. albus* on the basis of adhesion-defective strains that lacked specific surface proteins. The CBM37s were collectively shown to exhibit a broad specificity pattern, which indicated a mechanism for binding the parent enzymes to cellulosic substrates (Himmel et al., 2010). It is reported that CBM37 was responsible for anchoring substrate with enzymes to the cell surface. For example, CBM37 was reported as the mode that binds cellobiohydrolase and endoglucanase to the bacterial cell surface by the C terminus of glycoside hydrolases (Ezer et al., 2008). In addition, binding the substrate with enzymes (Himmel et al., 2010) might enhance the linkage between the cellulosome and enzymes related to the cellobiose degradation of *R. thermocellum* M3.

Meanwhile, CBM13 and CBM15 were also found in *R. thermocellum* M3. CBM13s acquire a larger variety of carbohydrate binding specificities including endo-β-glucanase (EC 3.2.1.6; Ferrer et al., 1996), α-galactosidase (EC 3.2.1.22; Holan et al., 1993), and some other glycoside hydrolases (White et al., 1995). Different from CBM13, CBM35 was reported to have conserved ligand specificity, which is often appended to plant cell wall-degrading enzymes (Montanier et al., 2009) and xylan-degrading enzymes (Chen et al., 1995), but it is often found in β-galactosidase (EC 3.2.1.23; Ali-Ahmad et al., 2017). Therefore, it is suggested that the β-galactosidase of strain M3 is mainly related to CBM13 and CBM35 modules during the degradation of cellulosic feedstocks.

### 4.3. Genome specificity of auxiliary activities of *Ruminiclostridium thermocellum*

AA6 and AA8 families were first identified in *R. thermocellum* in this study. In general, members of the AA8 family (cellobiose dehydrogenase, CDH) can be isolated or appended to a CBM. Proteins contain iron reductase domains and may generate reactive oxygen species that could contribute to the non-enzymatic degradation of cellulose chains by the generation of highly reactive hydroxyl radicals (OH•) *via* Fenton’s reaction (Levasseur et al., 2013). It is reported that the poor cellobiose availability of the substrate was a limiting factor to CDH activity; on the surface of *R. thermocellum*, cellobiose was accumulated in a restricted area, which was a sufficient substrate for the CDH. Good contact with CDH might play an important role in reducing the concentration of cellobiose in the environment (Kittl et al., 2012) and potentially releasing the strain M3 from the feedback inhibition of cellobiose.

## 5. Conclusion

The genome of *R. thermocellum* M3 harbored a high level of genomic uniqueness compared to other wild *R. thermocellum*. The majority of genes of *R. thermocellum* M3 fell into GHs and CBMs. Moreover, some unique genes were found in *R. thermocellum* M3 which were not found in *R. thermocellum*, which belong to the Auxiliary Activity Family (AA), cellulose-binding modules Family (CBM), Carbohydrate Esterase Family (CE), Glycosyl Transferases Family (GT), and Glycoside Hydrolase Family (GH). The hydrolysis of cellobiose by *R. thermocellum* M3 was conducted by the synergy of BglA and BglX, which not only hydrolyze cellobiose but also increase the affinity of Bgl for cellulosic feedstocks, increasing catalytic activity. Meanwhile, the GH42, GH116, CBM37, and AA8 families might participate in the cellobiose degradation that released the *R. thermocellum* M3 from the feedback inhibition of cellobiose.

## Data availability statement

The datasets presented in this study can be found in online repositories. The names of the repository/repositories and accession number(s) can be found in the article/Supplementary material.

## Author contributions

ST, MQ, LZ, SC, LiL, and LiuL contributed jointly to all aspects of the work reported in the manuscript. ST and LZ designed the experiment. MQ performed the experiments. ST, MQ, LiL, SC, and LiuL contributed to the data analysis. ST and MQ drafted the manuscript. All authors contributed to the article and approved the submitted version.

## Funding

This study was supported by the National Natural Science Foundation of China (No. 51908200), the Heilongjiang Provincial College Youth Innovation Talent Project (No. UNPYSCT-2020028), and the Basic Scientific Research Operating Expenses (No. 2021-KYYWF-1465).

## Conflict of interest

The authors declare that the research was conducted in the absence of any commercial or financial relationships that could be construed as a potential conflict of interest.

## Publisher’s note

All claims expressed in this article are solely those of the authors and do not necessarily represent those of their affiliated organizations, or those of the publisher, the editors and the reviewers. Any product that may be evaluated in this article, or claim that may be made by its manufacturer, is not guaranteed or endorsed by the publisher.

## Supplementary material

The Supplementary material for this article can be found online at: https://www.frontiersin.org/articles/10.3389/fmicb.2022.1079279/full#supplementary-material
