# Supplementary material for: Comparative genomics reveals cellobiose hydrolysis mechanism of Ruminiclostridium thermocellum M3, a cellulosic saccharification bacterium
Source: Front Microbiol. 2023 Jan 6;13:1079279. doi: 10.3389/fmicb.2022.1079279 (PMC9852859; doi:10.3389/fmicb.2022.1079279)
Supplement: Supplementary file 1 [file Data_Sheet_1.docx]

Supplementary Material

**Table S1** Gene annotation of *R. thermocellum* M3 using different databases.

| Strain | NR | KEGG | COG | GO | SwissProt | CAZy | CARD | VFDB | PHI |
| --- | --- | --- | --- | --- | --- | --- | --- | --- | --- |
| DSM 2360 | 2972 | 1234 | 2043 | 2043 | 2040 | 269 | 32 | 226 | 60 |
| ATCC 27405 | 3186 | 1225 | 2151 | 2151 | 2163 | 260 | 35 | 223 | 60 |
| DSM 1313 | 2944 | 1233 | 2037 | 2037 | 2041 | 273 | 32 | 228 | 60 |
| AD2 | 2965 | 1246 | 2042 | 2042 | 2038 | 269 | 32 | 227 | 60 |
| M3 | 3065 | 1246 | 2071 | 2021 | 2077 | 271 | 35 | 216 | 57 |

**Table S2** CRISPR arrays of *R.thermocellum* M3.

| Contig | Start | End | RepeatCount | RepeatLength | SpacerLength |
| --- | --- | --- | --- | --- | --- |
| Chrom_circularise | 844789 | 848505 | 51 | 37 | 36 |
| Chrom_circularise | 978094 | 987701 | 144 | 29 | 37 |
| Chrom_circularise | 1997899 | 2006882 | 134 | 30 | 37 |
| Chrom_circularise | 3516568 | 3522593 | 90 | 30 | 37 |

**Table S3** The statistical of gene distribution in gene islands of *R.thermocellum* M3.

| Number | GeneIsland | length | CG_Ratio(%) | Gene_Count |
| --- | --- | --- | --- | --- |
| GI1 | Chrom_circularise:904180-914473 | 10293 | 39.1042456 | 8 |
| GI2 | Chrom_circularise:874239-897835 | 23596 | 37.07408035 | 17 |
| GI3 | Chrom_circularise:642714-662304 | 19590 | 37.83052578 | 10 |
| GI4 | Chrom_circularise:3147450-3179405 | 31955 | 38.08480676 | 37 |
| GI5 | Chrom_circularise:1317295-1394521 | 77226 | 33.72957294 | 82 |
| GI6 | Chrom_circularise:2685889-2697164 | 11275 | 36.345898 | 9 |
| GI7 | Chrom_circularise:3033121-3045430 | 12309 | 35.21000894 | 12 |
| GI8 | Chrom_circularise:864545-871603 | 7058 | 34.44318504 | 8 |
|  | Total | 24162.75 | 36.47779043 | 183 |

**Table S4** Total genes concerning GH and CBM in *R. thermocellum* M3 genome.

| CAZy_family^1^ | Count | CAZy_family^1^ | Count | CAZy_family^1^ | Count |
| --- | --- | --- | --- | --- | --- |
| GH1 | 4 | GH51 | 1 | CBM6 | 17 |
| GH2 | 1 | GH53 | 1 | CBM11 | 2 |
| GH3 | 2 | **GH55** | 1 | CBM13 | 2 |
| **GH4** | 1 | **GH57** | 1 | CBM16 | 6 |
| GH5 | 13 | GH74 | 1 | CBM22 | 4 |
| GH8 | 2 | **GH76** | 2 | **CBM23** | 1 |
| GH9 | 17 | **GH78** | 5 | CBM25 | 3 |
| GH10 | 6 | **GH79** | 1 | CBM30 | 3 |
| GH11 | 2 | GH81 | 1 | CBM32 | 3 |
| GH13 | 2 | **GH85** | 1 | CBM34 | 1 |
| GH15 | 2 | **GH93** | 1 | CBM35 | 19 |
| GH16 | 2 | GH94 | 5 | **CBM36** | 11 |
| GH18 | 3 | **GH95** | 1 | **CBM37** | 1 |
| GH23 | 2 | **GH100** | 1 | **CBM40** | 1 |
| GH26 | 3 | **GH103** | 1 | CBM42 | 4 |
| **GH28** | 1 | **GH105** | 2 | CBM44 | 2 |
| GH30 | 2 | **GH106** | 1 | **CBM47** | 1 |
| **GH32** | 5 | **GH109** | 2 | CBM48 | 1 |
| **GH33** | 1 | **GH116** | 1 | CBM50 | 9 |
| **GH36** | 1 | **GH117** | 4 | **CBM53** | 2 |
| **GH37** | 1 | GH119 | 5 | CBM54 | 1 |
| GH39 | 3 | GH124 | 71 | **CBM61** | 4 |
| **GH42** | 2 | GH126 | 2 | CBM62 | 1 |
| GH43 | 7 | GH130 | 1 | **CBM70** | 4 |
| GH44 | 3 | GH133 | 2 | **CBM75** | 2 |
| **GH47** | 1 | CBM3 | 21 | **AA6** | 3 |
| GH48 | 2 | CBM4 | 5 | **AA8** | 1 |

1 Genes which not previously identified in *R. thermocellum* were marked in red and bold.

**Table S5** ABC-type sugar transport protein and cellobiose phosphorylase in *R. thermocellum* M3 genome.

| Gene ID^1^ | Gene | Description | Substrate range^2^ |
| --- | --- | --- | --- |
| **PROKKA_00080 PROKKA_01980** | MglA | ABC-type sugar transport system | Galactose Cellobiose Fructose  Arabinose |
| PROKKA_01296  PROKKA_01329 PROKKA_02765 PROKKA_02935 | UgpB | ABC-type sugar transport system | Maltose |
| **PROKKA_01330 PROKKA_02933** | UpgA | ABC-type sugar transport system | Trehalose  Cellobiose  Lactose  Arabinose Maltodextrin |
| PROKKA_01331 PROKKA_02931 PROKKA_02932 | UpgE | ABC-type sugar transport system | Monosaccharides  Polysaccharides |
| PROKKA_01086 PROKKA_02112 | Cbp | Cellobiose phosphorylase | Cellobiose |

1 ABC-type sugar transport system which transport cellobiose has been bolded**.** Genes which not previously identified in *R. thermocellum* are marked in red.

2 Date from NCBI Conserved Domains Database (CDD).


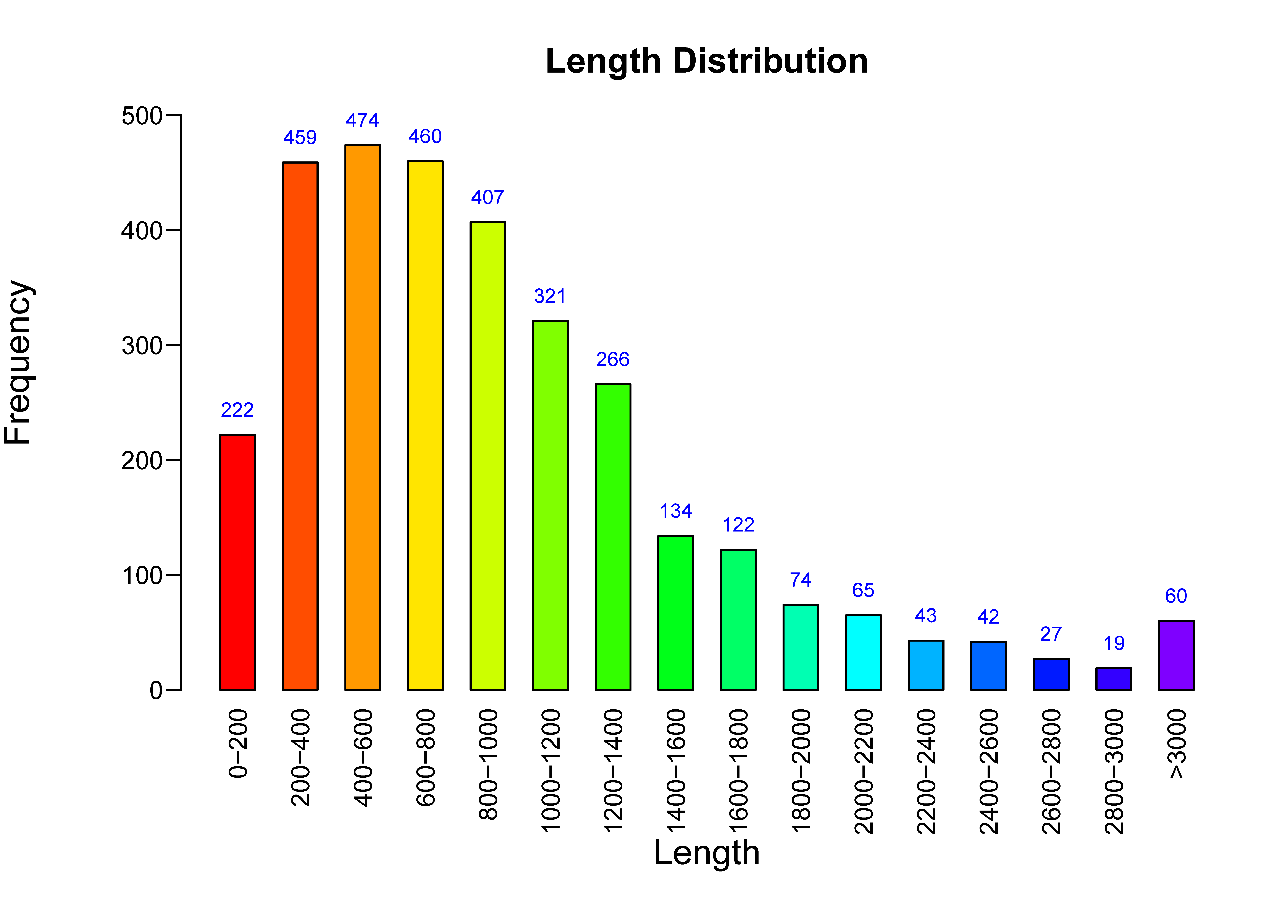


**Figure S1** Genome length distribution map of *R. thermocellum* M3


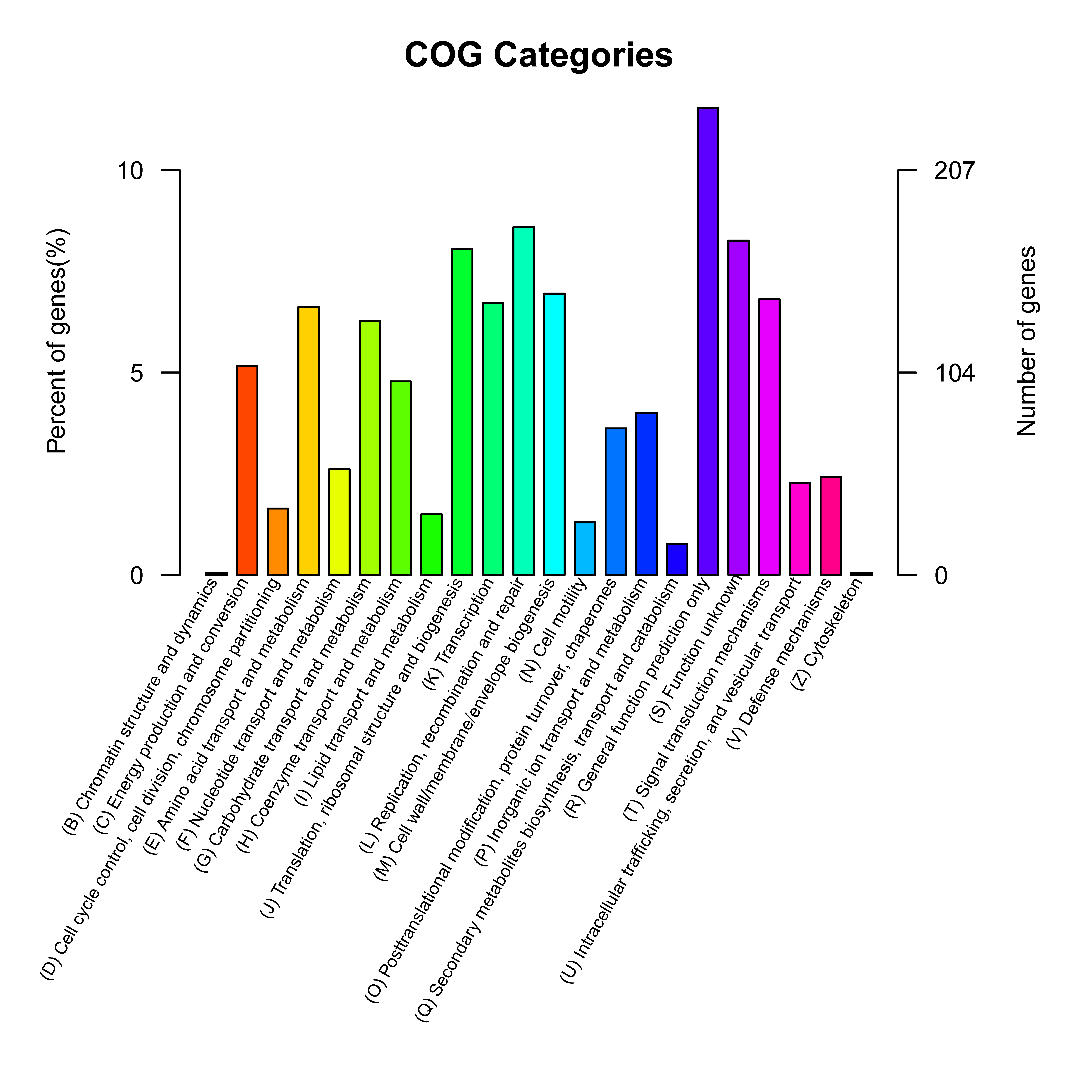


**Figure S2** Statistical map of functional annotation of *R. thermocellum* M3 using COG database

**
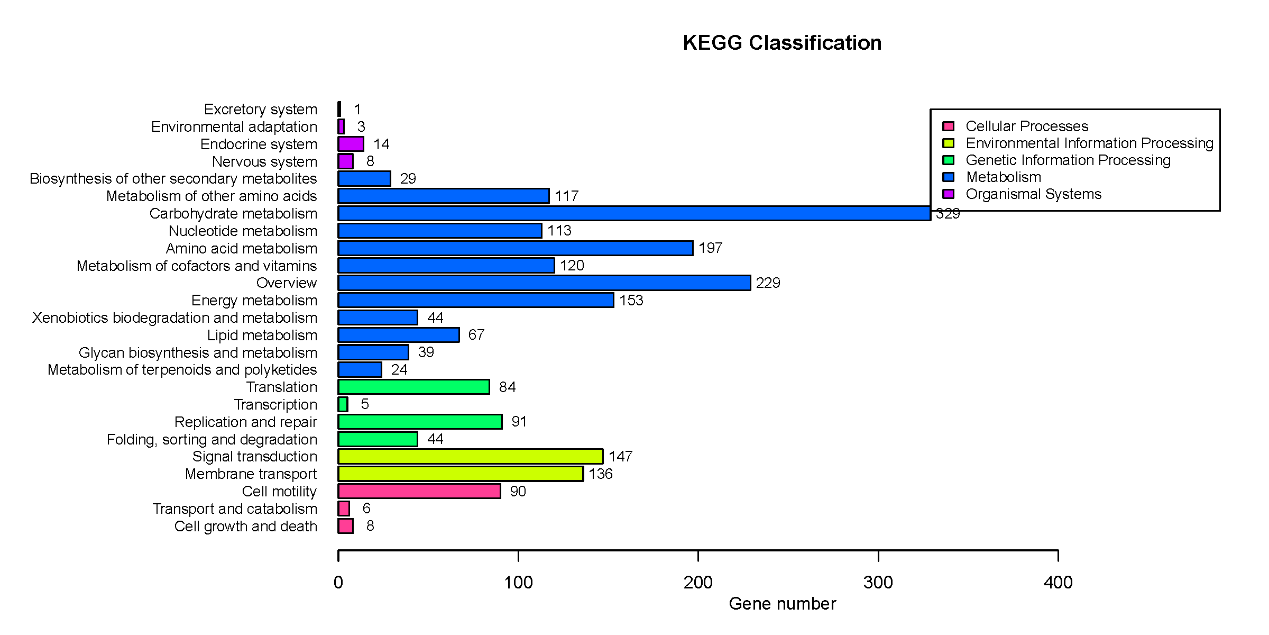
**

**Figure S3** The KEGG **(**Kyoto Encyclopedia of Genes and Genomes**)** metabolic pathway classification of *R. thermocellum* M3. The abscissa represents the number of annotated genes and the ordinate represents the metabolic pathway name.

**
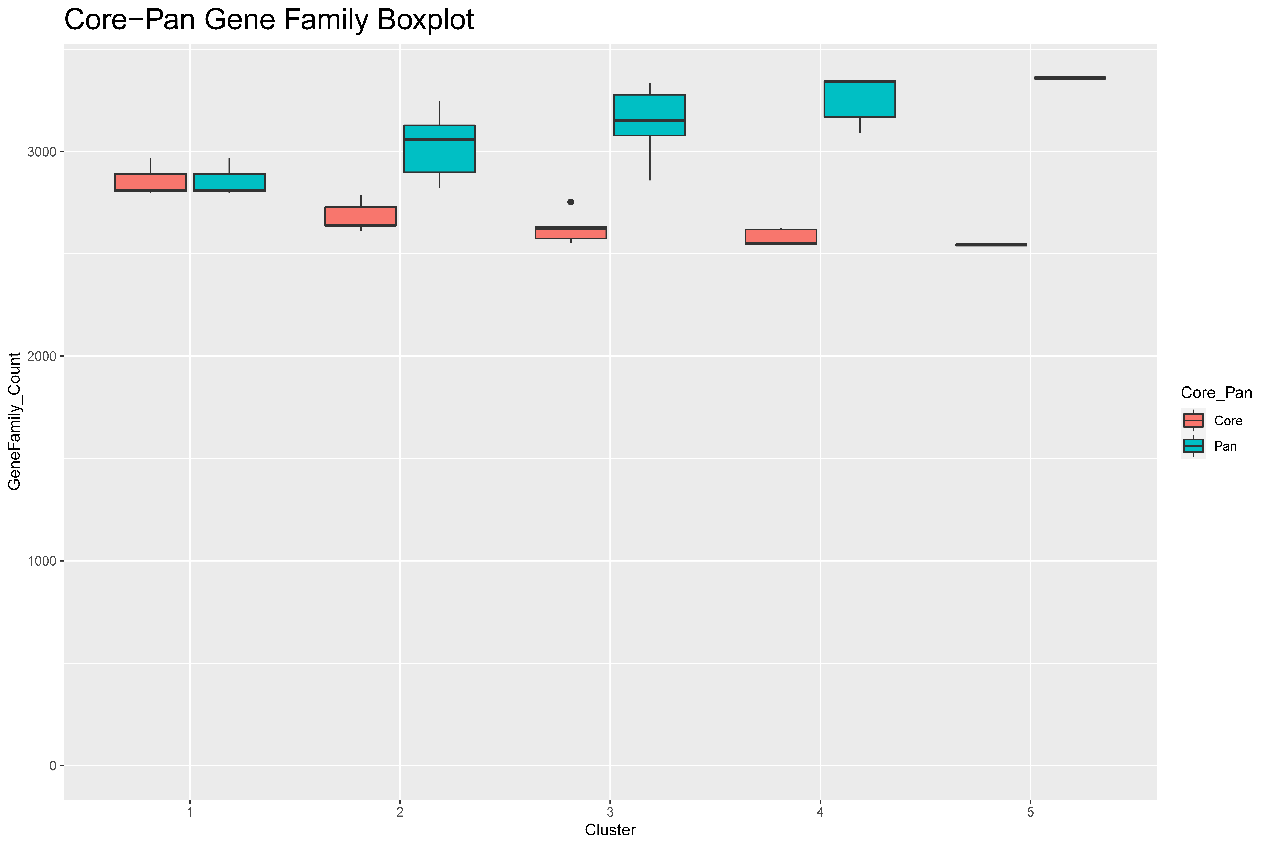
**

**Figure S4** Pan- and core-gene family boxplot of *R. thermocellum* based on the results of pan-genome analysis of 5 *R. thermocellum* strains

**
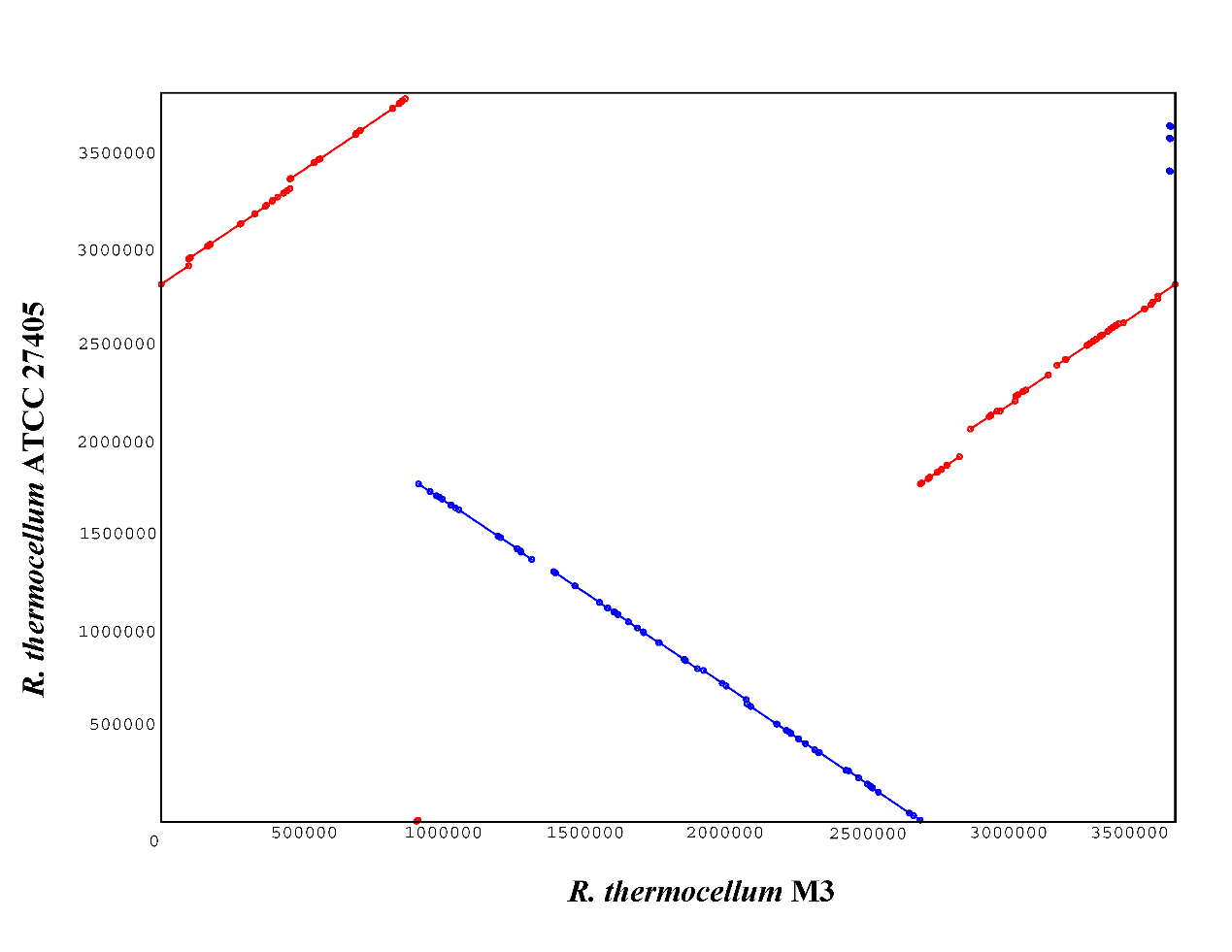
**

**Figure S5** Genome-wide comparison dot plot about *R. thermocellum* M3 and *R. thermocellum* ATCC 27405. Red: sequences are aligned in the same orientation; blue: sequences are aligned, but one is in the reverse complement orientation

**
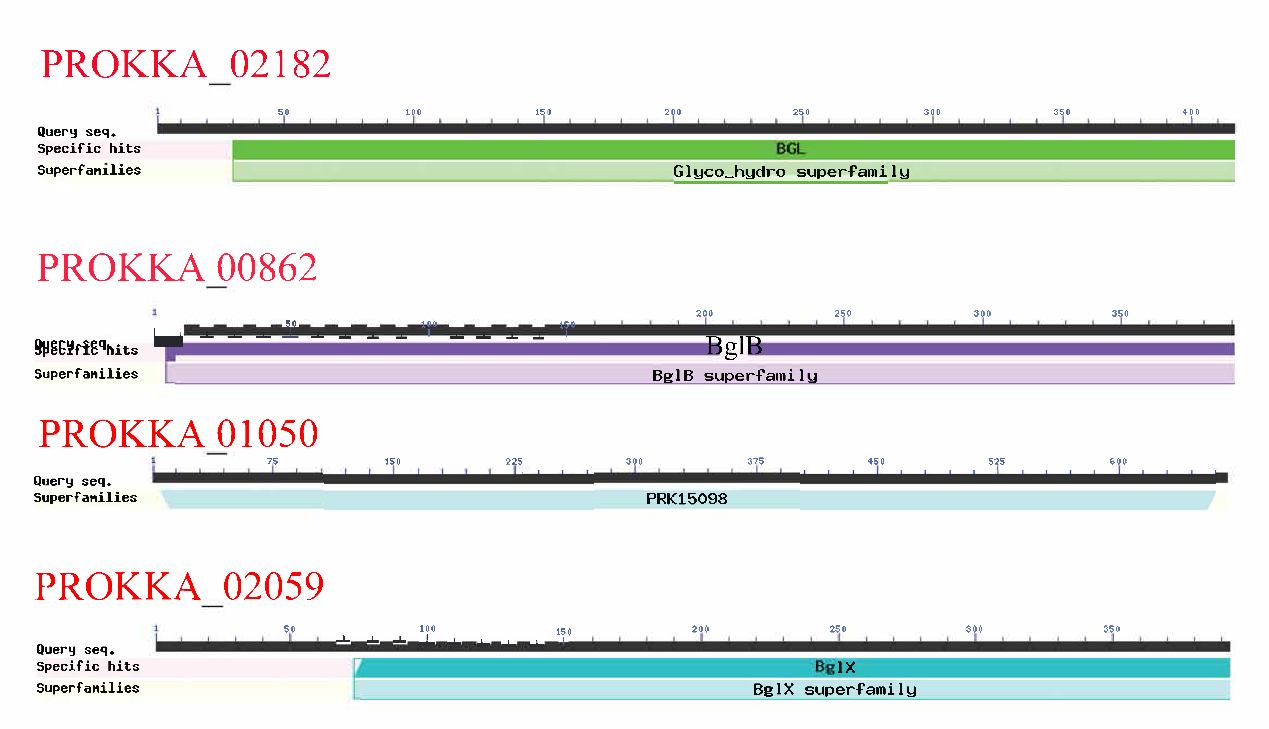
**

**Figure S6** The conserved domains prediction of β-glucosidase in *R. thermocellum* M3 genome. Putative conserved domains of four BGLs were detected by NCBI protein blast.
